# Clinical presentation and imaging findings in juvenile-onset back pain: a ten-year hospital-based retrospective analysis in Douala (Cameroon)

**DOI:** 10.3389/fped.2024.1424391

**Published:** 2024-07-02

**Authors:** Fernando Kemta Lekpa, Paul Eloundou, Jean Roger Moulion Tapouh, Sylvain Raoul Simeni Njonnou, Baudelaire Fojo Talongong, Francine Same Bebey, Estelle Megne Tamo, Diomède Noukeu, Dominique Enyama, Palma Haoua Abouame, Arielle Annick Sime Tchouamo, Henry Namme Luma, Madeleine Ngandeu-Singwe, Simeon Pierre Choukem

**Affiliations:** ^1^ Faculty of Medicine and Pharmaceutical Sciences, University of Dschang, Dschang, Cameroon; ^2^ Department of Internal Medicine, Douala General Hospital, Douala, Cameroon; ^3^ Health and Human Development (2HD) Research Network, Douala, Cameroon; ^4^ Faculty of Medicine and Pharmaceutical Sciences, University of Douala, Douala, Cameroon; ^5^ Faculty of Medicine and Biomedical Sciences, University of Yaoundé I, Yaoundé, Cameroon

**Keywords:** back pain, juvenile-onset back pain, children, pain, imaging, disc diseases, scoliosis

## Abstract

**Introduction:**

Studies exploring the clinical and imaging characteristics of juvenile-onset back pain (JOBP) are scarce. The purpose of this study was to assess the clinical presentation, imaging findings, and factors associated with JOBP.

**Methods:**

A retrospective record-based study was conducted among all pediatric cases presenting to the Rheumatology unit of the Douala General Hospital, Cameroon, from January 2014 to December 2023. The study did not include children whose back pain began after 16 years of age.

**Results:**

Of the 216 records of patients under 18 examined, 67 children (31 girls) were diagnosed with JOBP. The median age was 15 [13–16] years. More than two-third of the children included in this study had chronic pain (*n* = 46; 68.65%). Pain was mild to moderate in intensity in 48 children (71.6%). Radiculopathy was present in 10 children (14.92%). The most common location of back pain was the lumbar region (*n* = 64; 95.52%). Some children experienced pain in more than one location. The location of the pain was not associated with gender, duration of the pain, radiculopathy, or practice of a competitive sport (*p *> 0.05). Musculoskeletal abnormalities on imaging were found in 38 children (62.29%). In multivariate analysis, peripheral joint involvement [aOR = 0.253 (0.073–0.878); *p *= 0.030] and mild pain intensity [aOR = 0.077 (0.014–0.422); *p *= 0.003], were independently associated with the presence of musculoskeletal abnormalities on imaging.

**Conclusion:**

JOBP affects a third of children and adolescents of our clinic population. The common musculoskeletal abnormalities found on spine imaging are disc diseases and scoliosis.

## 1 Introduction

Back pain is one of the most widespread diseases of the musculoskeletal system and a leading global cause of years lived with disability (1, 2). Thought to be the prerogative of adults and elderly, back pain, particularly low back pain, is increasingly being described in children and adolescents (3, 4). Due to the heterogenicity of studies and the limited use of healthcare facilities, the true prevalence of juvenile-onset back pain (JOBP) in children and adolescents is not well-known (3). Its incidence and prevalence in Western countries are thought to be increasing in this age group. JOBP is much more frequent in the teenage years, with a high risk of conditions becoming chronic and might predict future back pain in adulthood (3–6).

The risk factors affecting the children include primarily the individual’s physical attributes and family history (7–9). In contrast to the long-held perception that JOBP is generally transient and insignificant in terms of impact on individuals, evidence suggests that it significantly impairs a substantial minority of children and adolescents who complain about it. This leads to absenteeism from school or work and/or missing out on sports or physical activities (3, 4, 6).

The available evidence recommends that every child with a complaint of back pain needs an extensive evaluation using advanced imaging and blood work to establish an underlying cause (3–5, 8, 10–13). The differential diagnosis of back pain in childhood is broad and different from that seen in adults. Back pain is labeled symptomatic or “specific” when there is an obvious etiology, particularly inflammatory, neoplastic or infectious conditions. It is commonly described as mechanical or “non-specific” (3–5, 8).

The previous African studies published on JOBP were surveys or cross-sectional studies performed in schools without medical imaging to obtain a differential diagnosis (9, 14–17). Only one study included clinical examination in its design, but no medical imaging was performed (9). Studies exploring JOBP's clinical and imaging characteristics in children have yet to be published in Cameroon. The purpose of this study was thus to assess the clinical presentation, presumed etiologies, and risk factors associated with JOBP among children and adolescents seen in a tertiary hospital in Cameroon. Our goal was to provide a better understanding of JOBP in African settings in order to inform management strategies.

## 2 Methods

### 2.1 Study design and setting

We conducted a retrospective record-based study involving pediatric patients seen over ten years, from January 2014 to December 2023. The study was performed at the Rheumatology outpatient clinic of the Douala General Hospital (DGH), Douala, Cameroon. The DGH is a referral centre and teaching hospital located in Douala, the economic capital of Cameroon, Central Africa. It receives patients from all the ten regions of Cameroon and neighboring countries, including Chad, the Central African Republic, Equatorial Guinea, and Nigeria. Cameroon has no pediatric Rheumatology clinic. Instead, children with rheumatic disease receive care from adult rheumatologists in collaboration with pediatricians at the hospital. Four adult rheumatologists were on duty during the study period.

### 2.2 Participants

Our study population consisted of all consenting consecutive outpatients with a recorded diagnosis of back pain, regardless of location. Back pain was defined as pain or discomfort in specific parts of the back, cervical (neck pain), thoracic (mid-back pain), or lumbar (low back pain) that was not due to trauma or menstrual pain (18). Back pain could be the primary or associated diagnosis during initial or subsequent visits. Children and adolescents included in this study had to be under 18. The study did not include those with back pain onset after age 16 (7). Traumatic etiologies were also excluded from this study. Then, JOBP was defined as any back pain that began before the age of 16. Medical records with incomplete information were also not included in the study**.**

### 2.3 Variables and data sources

Data during back pain diagnosis were collected on a standardized case-report form for each patient, including sociodemographic data, current and past medical history, clinical findings (pain, radicular pain, visual analogue scale), laboratory findings, and medical imaging data (spinal x-ray, CT-scan, and MRI).

### 2.4 Sample size

The sample size was calculated using the Cochrane formula. We used the prevalence of 12.3% obtained in a previous community-based study evaluating the prevalence of low back pain in schoolchildren in Cameroon (14). Thus, the minimum expected sample size was 166 children to be representative. However, we used a consecutive, non-probability sampling method to select eligible study participants. Patients with back pain who were treated at our outpatient clinic during the study period were identified through outpatient attendance books.

### 2.5 Data management and statistical methods

The data were collected and analyzed using SPSS version 23.0 software (Chicago, IL, USA). According to distribution, quantitative variables were summarized and presented as mean ± standard deviation (SD) or median [25–75th percentile]. Qualitative variables were summarized using absolute numbers and percentages (%). Statistical comparisons were made with the Student's *t*-test for continuous variables and the Chi-square test for categorical variables. All variables significantly associated with musculoskeletal abnormalities in univariate analysis were included in a multiple logistic regression model to adjust the confounding effects. The *p*-Value was considered significant for all these tests if it was less than 0.05.

## 3 Results

### 3.1 General characteristics of the study population

Two hundred and sixteen (216) outpatients aged 1–18 were seen in our clinic over the ten years. Of these, 69 patients had a JOBP diagnosis. Two patients were excluded because their back pain was trauma-related. Thus, we included 67 children (31.01%) aged 7–17 years with JOBP. Back pain was the primary diagnosis in 52 children and was considered an associated diagnosis in 15 children. At the time of diagnosis of JOBP, the median [25–75th percentile] age of participants was 15 [13–16] years. There were 31 girls and 36 boys, with a female: male ratio of 0.86:1. All the children attended school. Competitive sport was found in 13 children (all boys in our study), and four children had a previous diagnosis of juvenile idiopathic arthritis. Most of the children included in this study had chronic pain (*n* = 46; 68.7%). Pain was moderate to severe in 53 children (79.1%). Radicular pain was present in 10 children (14.9%). In these children with JOBP, peripheral joint involvement was predominant in the lower limbs, particularly the knees (*n* = 18; 26.86). Detailed characteristics of our study participants can be found in Table 1.

**Table 1:** Descriptive information of the study population.

Characteristics	All children (*n* = 67)	Gender		*p*-value
		Male (36)	Female (31)	
Demographics				
Age, years, median (IQR)	15 (13–16)	15 (12–16)	15 (13–16)	0.227
Age range, years				
6–8	3 (4.5)	2 (5.6)	1 (3.2)	1
8–10	4 (6.0)	2 (5.6)	2 (6.5)	1
10–12	7 (10.4)	4 (11.1)	3 (9.7)	1
12–14	9 (13.4)	2 (5.6)	7 (22.6)	0.070
14–16	32 (47.8)	19 (52.8)	13 (41.9)	0.464
16–18	12 (17.9)	7 (19.4)	5 (16.2)	0.761
Clinical data				
Location of back pain				
Cervical	3 (4.5)	1 (2.8)	2 (35.5)	0.592
Dorsal	29 (43.3)	15 (41.7)	14 (45.2)	0.809
Lumbar	64 (95.5)	34 (94.4)	30 (96.8)	1
Medical history				
Juvenile idiopathic arthritis	4 (6.0)	0	4 (6.0)	1
Cancer	1 (1.5)	0	1 (3.2)	1
Sickle cell disease	2 (3.0)	0	2 (6.5)	1
Leg length inequality	2 (3.0)	2 (5.6)	0	1
Obesity	1 (1.5)	0	1 (3.2)	1
Osgood-Schlater disease	1 (1.5)	1 (2.8)	0	1
Osteochondritis	2 (3.0)	2 (5.6)	0	1
Competitive sports	13 (34.3)	13 (36.1)	0	<0.001
Altered gait	1 (1.5)	1 (2.8)	0	1
Peripheral joints involvement				
Hip	4 (6.0)	2 (3.0)	2 (3.0)	1
Knee	18 (26.8)	9 (13.4)	9 (13.4)	1
Ankle	4 (6.0)	2 (3.0)	2 (3.0)	1
Foot	1 (1.5)	0	1 (1.5)	1
Shoulder	2 (3.0)	1 (1.5)	1 (1.5)	1
Elbow	4 (6.0)	1 (1.5)	3 (4.5)	0.586
Wrist	4 (6.0)	1 (1.5)	3 (4.5)	0.586
Duration of the back pain				
<3 week	12 (17.9)	5 (13.9)	7 (22.6)	0.355
3 week to 3 months	9 (13.4)	2 (5.6)	7 (22.6)	0.070
>3 months	46 (68.7)	29 (80.6)	17 (54.8)	0.024^a^
Radiculopathy				
Yes	10 (14.9)	3 (8.3)	7 (22.6)	0.168
No	57 (85.1)	33 (91.7)	24 (77.4)	
Visual analogue scale				
Mild	14 (20.9)	8 (22.2)	6 (19.4)	0.773
Moderate	34 (50.7)	20 (55.6)	14 (45.2)	0.396
Severe	19 (28.4)	8 (22.2)	11 (35.5)	0.282

^a^ OR = 3.412 (1.151–10.117).

Children aged 16–18 were likely to be involved in competitive sports [OR = 5.347 (2.187–13.072)]. Chronic pain was significantly more common in boys [OR = 3.412 (1.151–10.117); *p* = 0.024]. Radicular pain was significantly associated with pain severity [OR = 5.895 (1.699–20.448); *p* = 0.004], previous past medical history [OR = 5.2 (1.684–16.053); *p* = 0.006], and presence of disc diseases on imaging [OR =  4.667 (1.356–16.060); *p* = 0.019].

**Figure 1:**
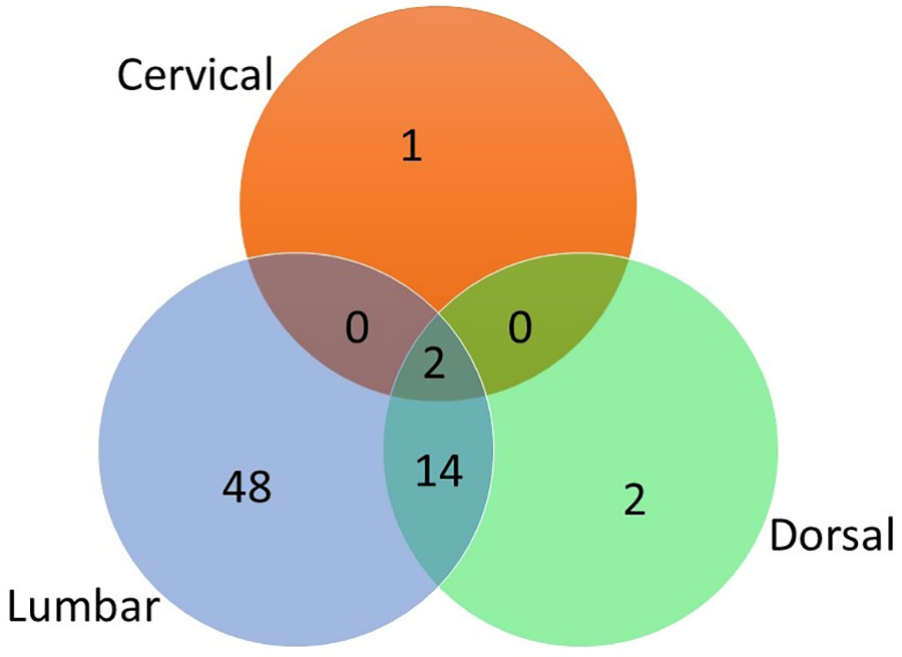
Venn diagram demonstrating the overlap between pain locations.

The main location of JOBP was the lumbar region (*n* = 64; 95.52%), followed by the thoracic region (*n* = 18; 26.86%) and the cervical region (*n* = 3; 4.47%). Some children experienced pain in more than one location (Figure 1). The location of the pain was not associated with gender, duration of the pain, radiculopathy, or practice of a competitive sport (*p* > 0.05).

### 3.2 Musculoskeletal abnormalities on imaging of the spine

Of the 67 participants with JOBP during the study period, 61 (91.04%) underwent imaging, including 61 plain spinal x-rays, 7 CT scans and 2 MRIs. Musculoskeletal abnormalities were found in 38 patients (62.29%) of these children. Four children had at least two abnormalities on imaging. Scoliosis (*n* = 9; 23.68%) and disc diseases (*n* = 10; 26.31%) were the prominent abnormalities (Table 2). Disc diseases included thinning disc (*n* = 8), disc herniation (*n* = 1) confirmed on CT scan, and bulging disc (*n* = 1). Scoliosis was isolated in six children, and associated in three children with disc disease, Scheuermann's disease and spondyloarthritis respectively. Four patients had specific causes of JOBP (spondyloarhtritis, spondylodiscitis, osteogenesis imperfecta, and vertebral metastasis).

**Table 2:** The musculoskeletal abnormalities found on imaging of the spine.

Underlying disease	Imaging findings	*n* (%)
Non specific	No abnormalitie	29 (43.28)
	Disc diseases	10 (26.31)
	Scoliosis	9 (23.68)
	Spondylolysis	7 (18.42)
	Lumbosacral transitional vertebrae	4 (10.52)
	Scheuermann's kyphosis	3 (7.89)
	Spina bifida occulta	3 (7.89)
	Spondylolisthesis	3 (7.89)
	Lumbar spinal stenosis	1 (2.63)
Specific	Spondyloarthritis	1 (2.63)
	Spondylodiscitis	1 (2.63)
	Osteogenesis imperfecta	1 (2.63)
	Vertebral metastasis	1 (2.63)

In univariate analysis (Table 3), factors significantly (*p* < 0.05) associated with imaging findings were peripheral joint involvement, mild pain intensity, and pain duration [acute pain (<3 weeks) or chronic pain (>3 months)]. However, in multivariate analysis, peripheral joint involvement [ORa = 0.253 (0.073–0.878); *p* = 0.030] and mild pain intensity [ORa = 0.077 (0.014–0.422); *p* = 0.003], were independently associated with the presence of musculoskeletal abnormalities on imaging (Table 4).

**Table 3:** Factors associated with musculoskeletal abnormalities found on imaging of the spine in univariate analysis.

Characteristics	Abnormalities in spinal imaging		*p*-value
	Yes	No	
Gender			
M	23 (60.5)	13 (44.8)	0.226
F	15 (39.5)	16 (55.2)	
Age range, years			
6–8	2 (5.3)	1 (3.4)	1
8–10	0	4 (13.8)	0.031
10–12	2 (5.3)	5 (17.2)	0.225
12–14	5 (13.2)	4 (13.8)	1
14–16	22 (57.9)	10 (34.5)	0.057
16–18	7 (18.4)	5 (17.2)	0.901
Peripheral joint involvement			
Yes	8 (21.1)	13 (44.8)	**0**.**038**
No	30 (78.9)	16 (55.2)	
Location of pain			
Cervical	2 (5.3)	1 (3.4)	1
Thoracic	13 (34.2)	16 (55.2)	0.086
Lumbar	36 (94.7)	28 (96.6)	1
Intensity of pain			
Mild	2 (5.3)	12 (41.4)	**<0.001**
Moderate	13 (34.2)	12 (41.4)	0.180
Severe	36 (94.7)	5 (17.2)	0.078
Radiculopathy			
Yes	8 (21.1)	2 (6.9)	0.168
No	30 (78.9)	27 (93.1)	
Duration of pain			
<3 weeks	3 (7.9)	9 (31.0)	**0**.**014**
3 weeks to 3 months	4 (10.5)	5 (17.2)	0.485
>3 months	31 (81.6)	15 (51.7)	**0**.**009**

Significant statistical values are shown in bold.

**Table 4:** Multivariate regression model showing factors independently associated with musculoskeletal abnormalities on imaging in patients with back pain.

Variables	Crude OR (95%CI)	Crude *p-*value	Adjusted OR (95%CI)	Adjusted *p-*value
Peripheral joint involvement (Yes/No)	0.328 (0.113–0.956)	0.038	***0.253*** (***0.073–0.878)***	***0***.***030***
Pain intensity (mild)	0.079 (0.016–0.391)	<0.001	***0.077*** (***0.014–0.422)***	***0***.***003***
Pain duration (<3 weeks)	0.190 (0.046–0.786)	0.014	0.424 (0.053–3.388)	0.419
Pain duration (>3 months)	4.133 (1.380–12.379)	0.009	1.983 (0.390–10.086)	0.409

Significant statistical values are shown in bold.

## 4 Discussion

In a ten-year retrospective record-based study of children and adolescents seen in a Rheumatology clinic in Cameroon, we found that JOBP, particularly low back pain, was common. The presence of musculoskeletal abnormalities detected on imaging was negatively correlated with the pain's intensity and peripheral joint involvement.

We found that around one-third of children and adolescents had JOBP. This prevalence is comparable to that reported in a specialized Rheumatology clinic in Spain (19), and in a community study in Denmark (20). As expected, competitive sport was associated with JOBP, particularly in boys (3–8). However, in the absence of girls practicing a competitive sport in our study, we cannot link risk factors to the occurrence of musculoskeletal abnormalities on imaging. JOBP was not isolated in our patients as a painful skeletal location. As in the literature, at least two pain sites were commonly involved (20, 21). Contrary to expectations, peripheral joint involvement reduced the likelihood of musculoskeletal abnormalities on imaging in patients with JOBP in our study. This result could be supported by the fact that limb involvement would suggest etiologies such as juvenile idiopathic arthritis or other peripheral arthritis, with back pain perceived as referred pain. However, the notion of “multi-site bodily”, defined as the presence of at least two painful sites, would increase the likelihood of back pain (21). This discrepancy could be explained by the design of our study, with probably missing data on limb involvement reported in the children's medical records.

Published data suggest that most cases of JOBP in children are “non-specific”, musculoskeletal and self-limiting (3–5, 10). Consistent with previous studies (10), the etiology of JOBP was “non-specific” in most of our cases. However, pain was not self-limiting, as more than two thirds of our children had chronic pain. This could be explained by the fact that the patients seen in our clinic are generally those with the most severe conditions. Nevertheless, this is a warning sign because the presence of chronic pain (almost comparable to a constant pain) predicts the risk of suffering from a specific diagnosis, whether or not associated with other predictors like night pain, radicular pain, and abnormal neurological examination. Indeed, the predicted probability of having a specific diagnosis was 100% when a patient had three of the predictors, 85.7% for two predictors, 61.1% for one predictor, and 18.6% for zero predictors (11). Without any trauma, radicular pain, which is less frequent in our study than in the literature (10), is suggestive of disc disease (10, 11, 13). As with disc disease, we also observed cases of scoliosis in proportions comparable (11) or even higher (10) than those described in previous studies. However, there is conflicting evidence regarding the relationship between back pain and scoliosis (22, 23). More than a third of patients with adolescent idiopathic scoliosis may have spinal abnormalities on MRI (22). This frequency could be higher in our study, as one-third of our patients had an abnormality on spinal x-ray alone.

To the best of our knowledge, this is the first study to present a panoramic view of the clinical characteristics (obtained after a comprehensive clinical examination summarized in the medical record) and presumed etiologies (after spine imaging) of JOBP in children and adolescents in an African setting. Although not exhaustive, the imaging findings observed in this study are broadly in line with the data available, ranging from “evil” etiologies such as spondylodiscitis or neoplasia to “non-specific” etiologies (3, 4, 7, 24, 25). Despite this significant strength of our study, there are several limitations. First, the cross-sectional design of our study does not allow us to assert a causal relationship between imaging findings and JOBP. It is, therefore, not always possible to distinguish etiological factors from prognostic factors (6). Furthermore, we cannot formally exclude the existence of possible confounding factors in our sample (6). Secondly, retrospective data collection limits the generalizability of our results. Numerous missing data, such as disability and quality of life scores, psychological impact and school absenteeism related to back pain, would have improved the quality of this study. Cameroon is a low-income country with limited access to specialist care at an affordable cost to the community, so medical imaging could not be obtained for all patients. Some patients who had benefited from a spinal x-ray and needed a CT scan or MRI, as suggested by certain recommendations (11–13), could not do so. Bone scintigraphy, recommended in some diagnostic algorithms (11, 13) for the managing JOBP, is unavailable in Cameroon. This limitation can be put into perspective because most of the abnormalities found in our study were benign. Also, certain abnormalities found on imaging would not explain the back pain. In fact, in a recent study aimed at determining the natural history of disc changes from childhood to early adulthood and the possible association of these changes with low back pain, the authors showed that changes in the intervertebral disc signal on MRI were not associated with the presence of low back pain in childhood, adolescence and adulthood (26). However, MRI is increasingly used as a first-line imaging modality when at least one red flag is present and severe pathology is suspected in a child with back pain (11, 25). There is also an ambiguous relationship between scoliosis and back pain. The evidence suggests no direct relationship exists between the spine's deformity magnitude and back pain intensity (23). Thirdly, the expected minimum sample size of 166 children was not achieved. A larger sample size would have been desirable. However, it should be remembered that only 216 children and adolescents have been seen in the last ten years in our clinic. Hence, there is a need to consider carrying out a multicentre prospective study in an African setting. This sample size could have been larger if the study had been conducted in a pediatric Rheumatology clinic. This would require a greater interest in Rheumatology in Africa and a greater commitment to pediatric Rheumatology (27, 28). To date, despite the growing interest in pediatric Rheumatology in Cameroon, there is no Rheumatology clinic dedicated to the pediatric population in the country.

Despite the limitations of this study, the 67 participants with JOBP recruited over ten years represent a first in Africa. Although broadly similar to previously published data, our study helps to show that back pain exists in African children and adolescents. The dissemination of these results could guide caregivers and clinicians in Africa in the diagnostic and therapeutic approach to JOBP. This would be easier to implement if policymakers include JOBP as a public health problem, as we hope they will for back pain in adults. Also, it will enable to outline the importance of enhancing care coordination among the interprofessional team to ensure proper evaluation and management of JOBP. Then, the available data should lead us to adapt existing diagnostic algorithms (8, 11–13) and recommendations for the preventive and curative management of JOBP (8) to the specificities of Africa. These expected algorithms, adapted to African specificities, will gain power when updated following additional studies using a more rigorous design. These studies could be used to confirm our results and also to look for a causal link between the abnormalities observed on imaging and JOBP.

In conclusion, JOBP affects a third of the children and adolescents seen in a Rheumatology clinic in Cameroon. The clinical presentation is classic, but in more than two-thirds of children are seen with chronic pain of severe to moderate intensity. Although not exhaustive, musculoskeletal abnormalities found on spinal imaging were dominated by disc diseases and scoliosis. Despite the study's limitations, particularly those associated with the sample size, these data help show that JOBP exists in pediatric Rheumatology in African context, where it could constitute a real public health problem. This is a call to caregivers and policymakers to take action to create pediatric Rheumatology clinics and to organize holistic management of JOBP according to African specificities. However, further studies with robust design are needed to reduce the impact of our study's limitations.

## Data Availability

The original contributions presented in the study are included in the article/Supplementary Material, further inquiries can be directed to the corresponding author.
